# Application of Rice-Straw Biochar and Microorganisms in Nonylphenol Remediation: Adsorption-Biodegradation Coupling Relationship and Mechanism

**DOI:** 10.1371/journal.pone.0137467

**Published:** 2015-09-08

**Authors:** Liping Lou, Lingdan Yao, Guanghuan Cheng, Lixiao Wang, Yunfeng He, Baolan Hu

**Affiliations:** College of Environmental & Resource Sciences, Zhejiang University, Hangzhou, China; CAS, CHINA

## Abstract

Biochar adsorption presents a potential remediation method for the control of hydrophobic organic compounds (HOCs) pollution in the environment. It has been found that HOCs bound on biochar become less bioavailable, so speculations have been proposed that HOCs will persist for longer half-life periods in biochar-amended soil/sediment. To investigate how biochar application affects coupled adsorption-biodegradation, nonylphenol was selected as the target contaminant, and biochar derived from rice straw was applied as the adsorbent. The results showed that there was an optimal dosage of biochar in the presence of both adsorption and biodegradation for a given nonylphenol concentration, thus allowing the transformation of nonylphenol to be optimized. Approximately 47.6% of the nonylphenol was biodegraded in two days when 0.005 g biochar was added to 50 mg/L of nonylphenol, which was 125% higher than the relative quantity biodegraded without biochar, though the resistant desorption component of nonylphenol reached 87.1%. All adsorptive forms of nonylphenol (*f*_*rap*_, *f*_*slow*_, *f*_*r*_) decreased gradually during the biodegradation experiment, and the resistant desorption fraction of nonylphenol (*f*_*r*_) on biochar could also be biodegraded. It was concluded that an appropriate amount of biochar could stimulate biodegradation, not only illustrating that the dosage of biochar had an enormous influence on the half-life periods of HOCs but also alleviating concerns that enhanced HOCs binding by biochar may cause secondary pollution in biochar-modified environment.

## Introduction

Compared with conventional carbonaceous materials such as commercial activated carbon, the primary advantages of biochar are its low cost and the diversity of biomass from which it can be produced [1]. Because of its strong adsorption capacity, biochar has attracted increasing interest in recent years for the immobilization of HOCs [2–4] and is a promising solution in remediation [5,6]. Therefore, investigating the influence of biochar on the migration and transformation of HOCs is important for HOCs pollution control and eco-environmental security.

In previous studies, it has been demonstrated that biochar application has significant negative effects on HOCs degradation [7,8]. On one hand, the polyaromatic recalcitrant matrix of biochar is highly stable, and biochar may remain in the environment for tens or hundreds of years [9,10]. On the other hand, biochar has been shown to reduce the concentration of aqueous HOCs by reactive surface adsorption or physical trapping within biochar pores [11,12]. For the above two reasons, the desorption efficiency of adsorbed HOCs is very slow [13,14], resulting in reduced biodegradability and poor bioavailability [15–17]. From the above findings, it was proposed that biochar application suppressed the transformation of HOCs, resulting in a longer half-life and persistence in the soil and/or sediment. For instance, Muter et al. investigated the influence of biochar on the persistence of the herbicide 4-chloro-2-methylphenoxyacetic acid (MCPA) in soils and found that after 37 days, the biodegradation rate was significantly reduced when 5.3% biochar was applied, dropping from 100% to 69.3% (*P* < 0.01) [17]. A similar phenomenon was also observed by Xin et al., who found that the transformation rate of 2,2’,4,4’-tetrabromodiphenyl ether was decreased by 87.50–92.19% when 1% biochar was added to the soil [18].

Despite extensive data showing the reduced bioavailability of contaminants in the presence of biochar, other studies investigating HOCs biodegradation have indicated that biochar application promotes HOCs mineralization in the presence of abundant microorganisms [19–21]. Biochar is typically prepared at temperatures ranging from 300–700°C, thus biochar is rich in nutrient and trace elements (e.g., P、C、Na and Mo) for biota [22,23], which gives biochar the potential to have a positive impact on contaminant transformation [21] or no significant adverse effects [24]. Tong et al. found that 1% biochar accelerated PCP transformation from 12.5% to 60.7%, and 100% transformation rate was observed when a 5% dosage of biochar was added [21]. A possible explanation may be that the microorganisms were capable of adhering to or forming biofilms on biochar particles by releasing extracellular enzymes, which constitutes an important mechanism to overcome mass-transfer limitations in environmental remediation [25,26].

There is no consensus regarding the complex interactions resulting from biochar application due to variations in experimental time scale, HOCs concentration, biochar dosage etc. Therefore, it was speculated that there may be an appropriate biochar dosage range to enhance biodegradation rather than inhibit it, thus optimizing coupled adsorption-biodegradation for a specific concentration of HOCs. To verify these conjectures, nonylphenol, a representative HOCs with estrogenic effects, was selected as the target contaminant, while rice-straw-derived biochar was selected as an adsorbent. First, the effects of biochar dosage on the adsorption-biodegradation of various concentrations of nonylphenol were investigated. Second, changes in the adsorptive form of nonylphenol during biodegradation were investigated using Tenax desorption technology, and the biodegradation potential of the desorbed forms of nonylphenol was also calculated. Third, the coupled effects of biochar and microorganisms on the fate of HOCs were discussed.

## Materials and Methods

### Chemicals and materials

Nonylphenol (> 99% purity) was purchased from Aladdin (Shanghai, China) and prepared as a concentrated stock solution with acetonitrile. Tenax TA (60–80 mesh) was obtained from Supelco (Bellefonte, Pennsylvania, USA) and regenerated by ultrasonic washing with methanol, acetone and hexane in order [14]. Acetonitrile, methanol, acetone, hexane and dichloromethane (chromatographic grade) were purchased from Sigma-Aldrich (St. Louis, MO, USA).

Biochar was produced from rice straw following a procedure detailed in a previous study [14].

### Preparation of nonylphenol biodegradation inoculum

A mixed cultivation inoculum, able to utilize nonylphenol as a sole carbon source for growth, was isolated from sediments (0–10 cm depth) collected from the Qiantang River in Hangzhou, Zhejiang Province, China. A mineral salt medium was prepared containing (per liter) 4.0 g of K_2_HPO_4_, 4.0 g of NaH_2_PO_4_, 2.0 g of (NH_4_)_2_SO_4_, 0.41 g of MgSO_4_·7H_2_O, 0.01 g of anhydrous CaCl_2_, 0.01 g of MnSO_4_·H_2_O, and 0.01 g of FeSO_4_·7H_2_O, pH was maintained at 7.0–7.2 using 1 M NaOH. A 25-g sediment sample was added to a 250-mL flask containing nonylphenol-spiked medium (90 mL medium with 10 mg/L nonylphenol). The flask was incubated at 30°C on a 150 rpm shaker in the dark for 7 days, after which 10 mL of the liquid was transferred to another flask with nonylphenol-spiked medium (20 mg/L) and incubated under the same conditions. Successive incubations in nonylphenol-spiked medium (30, 40, 50 mg/L) were performed until colonies showed evidence of nonylphenol degradation.

The culture was enriched in medium (1 L medium contained 5.0 g of NaCl, 10.0 g of tryptone, and 5.0 g of beef extract, pH was maintained at 7.0–7.2 using 1 M NaOH) at 30°C while shaking at 150 rpm. After 24 h incubation, the culture was centrifuged at 4000 g for 10 min. The supernatant was discarded, and the microorganisms were resuspended in mineral salt medium. This procedure was repeated four times to ensure a thorough removal of any residual nutrient substances. The OD_600_ was adjusted to 1.0, and the inoculum was stored in refrigerator at 4°C until adding to the reactor.

### Pre-adsorption experiments

All sorption experiments were conducted in triplicate. The batch reactors consisted of 50-mL glass centrifuge vials with permeable silica gel stoppers to allow adequate oxygen. Each reactor contained 9 mL mineral salt medium with 0, 0.005, 0.01 or 0.10 g biochar, and all reactors were autoclaved twice. Nonylphenol stock solution with concentration of 1000 mg/L was added with 6, 30, 50 and 100 μL, respectively, to each vial to obtain final concentrations of 6, 30, 50, and 100 mg/L, and vials were then shaken at 150 rpm and 30°C in an orbital shaker for 24 h to reach adsorption equilibrium.

### Nonylphenol biodegradation experiments

Nonylphenol biodegradation experiments were performed in triplicate. A 1-mL aliquot of prepared nonylphenol biodegradation inoculum was added to all reactors, and the reactors were shaken at 150 rpm on a horizontal shaker at 30°C for 0, 1, 2, 4, 6, 10 and 16 d in the dark. Both the aqueous and solid concentrations of nonylphenol were measured. The sum of these quantities was defined as the residual. The concentration suppression curve of nonylphenol was explored using equal biochar dosages and a series of nonylphenol concentrations, using an identical set-up except that the sampling time was 6 d. To quantify the loss of nonylphenol due to abiotic processes and systematic loss, sterilized reactors with 0.2 mg/mL sodium azide were operated in parallel.

#### Analysis of aqueous nonylphenol concentration

At each designated time, reactors were taken from the shaker and centrifuged at 4000 g for 10 min to separate the aqueous and solid phases. In this study, the aqueous phase means the freely dissolved form in the mineral salt medium, and the solid phase is the form sorbed by biochar and/or microorganisms. From the aqueous phase, 0.5-mL aliquots were transferred to a 2-mL plastic centrifuge tube, and methanol was added at a water/methanol volumetric ratio of 1:1 to dissolve nonylphenol. Samples were then analyzed by high performance liquid chromatography (Agilent 1100 series) using a previously reported method [14].

#### Analysis of solid phase nonylphenol concentration

The supernatant was discarded, after which a defined amount of anhydrous sodium sulfate was added to the precipitate, and nonylphenol was extracted with 5 mL of organic extractant (methanol:methylbenzene = 6:1, v/v) by sonication for 40 min. The extracts were collected into nitrogen blowpipes. This procedure was repeated four consecutive times to ensure thorough extraction of the nonylphenol from the sodium sulfate. Eventually the collected solvent was nitrogen-flushed to 1 mL and analyzed by high performance liquid chromatography.

### Desorption of residual nonylphenol after degradation

The experimental set-up was the same as in the biodegradation experiments mentioned above. At each designated time interval, vials were taken from the shaker and 0.2 mg/mL sodium azide was added to stop biodegradation. Then, 0.1 g of Tenax beads and another 20 mL of mineral salt medium were added to begin the desorption experiment. The subsequent steps have been detailed in a recent work [14]. Finally, the combined extracts from the Tenax beads were concentrated to 1 mL using nitrogen flow and analyzed by high performance liquid chromatography.

### Data analysis

#### Concentration suppression curve of nonylphenol

The rate of nonylphenol biodegradation was plotted against time (t), and the Haldane model was fitted to the data, allowing the optimal concentration of nonylphenol to be calculated:

V=Vmax1+KmS+SKi (1)

$$S_{\max}=\sqrt{K_{m}K_{i}}$$ (2)

where *V* represents the substrate reaction rate (i.e., the nonylphenol biodegradation rate in this study), *V*_*max*_ represents the maximum reaction rate, *K*_*m*_ represents the half rate constant, *K*_*i*_ represents the inhibition constant, *S* represents the substrate concentration, and *S*_*max*_ represents the optimal concentration.

#### Desorption data interpretation

The initial adsorbate concentration in the system was assumed to be *S*_*0*_, and the residue after biodegradation time *T* was *S*_*T*_. Kinetic Tenax desorption experiments were preformed after the nonylphenol biodegradation step, and the adsorbate concentration at desorption time *t* was *S*_*t*_. Nonylphenol desorption data after biodegradation were fitted by the modified two-domain model using Origin 8.0 software [14].

StST=Frape−krapt+Fslowe−kslowt+Fr (3)

where *F*_*rap*_, *F*_*slow*_ and *F*_*r*_ represent the rapid, slow and resistant fractions, respectively, and *F*_*rap*_+*F*_*slow*_+*F*_*r*_ = 1; *k*_*rap*_ and *k*_*slow*_ represent the kinetic constants (h^-1^) of each domain.

By multiplying both sides of the equation by *S*_*T*_/*S*_*0*_,

StS0=frape−krapt+fslowe−kslowt+fr (4)

$$f_{rap}=F_{rap}\cdot \frac{S_{T}}{S_{0}}$$ (5)

$$f_{slow}=F_{slow}\cdot \frac{S_{T}}{S_{0}}$$ (6)

$$f_{r}=F_{r}\cdot \frac{S_{T}}{S_{0}}$$ (7)

where *f*_*rap*_, *f*_*slow*_ and *f*_*r*_ represent the three fractions of the transmutative two-domain model beginning with biodegradation.

### QA/QC

All experiments treatments were in triplicate, sterilized reactors with 0.2 mg/mL sodium azide were included as control. Pure acetonitrile: methanol (9:1, v/v), the mobile phase for HPLC analysis was analyzed in HPLC as solvent blank. From preliminary experiments, the average extraction recovery of nonylphenol samples was greater than 98% for biodegradation experiments. Statistical analysis of data was analyzed with Origin 8.0 and SPSS 11.0. Significance was assigned at *P* ≤ 0.05.

## Results and Discussion

### Effect of biochar on nonylphenol biodegradation

**Fig 1:**
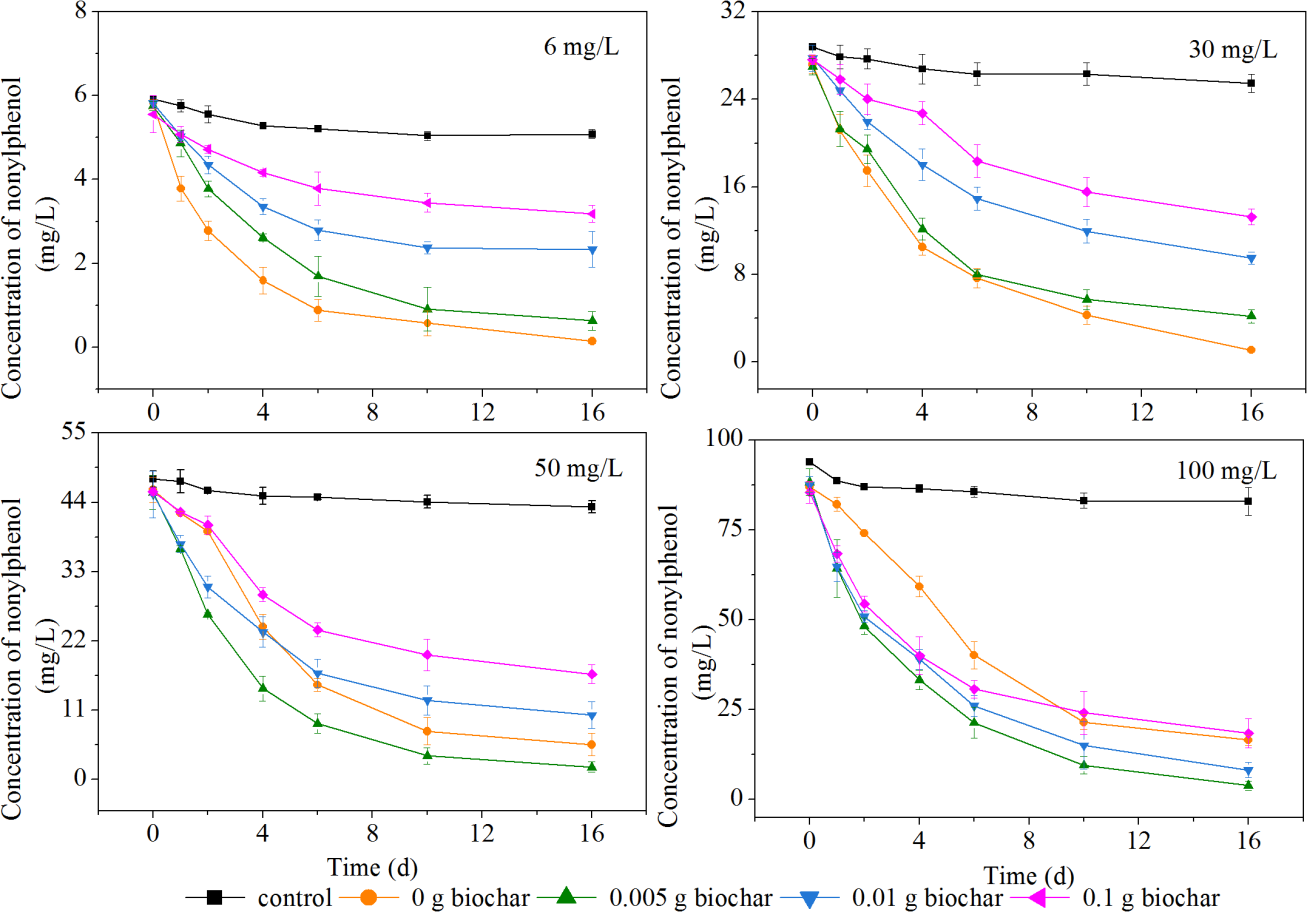
Changes in nonylphenol concentration with different biochar dosages. The degradation kinetics of different concentrations of nonylphenol (6, 30, 50 and 100 mg/L) at various biochar dosages (0, 0.005, 0.01 and 0.1 g).

Two mechanisms resulted in the disappearance of nonylphenol: abiotic dissipation and biotic degradation. Control reactors representing abiotic loss showed no significant reduction in nonylphenol (*P* > 0.05), indicating that biodegradation was responsible for the transformation of nonylphenol. For all experimental groups, nonylphenol underwent progressive reduction over 16 d of incubation, as shown in Fig 1.

To identify the role of biochar in nonylphenol biodegradation at various initial concentrations of pollutant, the nonylphenol concentrations were divided into low (*C*_*0*_ = 6 and 30 mg/L) and high (*C*_*0*_ = 50 and 100 mg/L) ranges. In the absence of biochar, biodegradation of nonylphenol was found to be generally efficient from an initial 6 mg/L to a final 0.574 mg/L, with a biodegradation rate of 90.4% over 16 d of incubation; this rate was similar to previously reported results [27–29]. Therefore, observations from this and other studies suggest that biodegradation may be an important method to govern HOCs such as nonylphenol [30,31]. However, the residual nonylphenol was significantly greater with biochar addition (*P* < 0.01), and the higher the dosage of biochar, the more the residual nonylphenol increased (*P* < 0.01). In particular, at a dosage of 0.1 g, almost half of the nonylphenol was not transformed, indicating that biochar application inhibited nonylphenol biodegradation at a low nonylphenol concentration. When more biochar was added, there was an enhanced negative effect on nonylphenol transformation. Similar phenomena have been observed in other studies as well [17,32]. Biochar is widely regarded as an effective adsorbent to adsorb a broad range of HOCs due to its large surface area and highly aromatic structure [11]. Nevertheless, this high capacity for sorption may decrease the bioavailability and reactivity of contaminants, resulting in poor biodegradability [16,33].

For the high nonylphenol concentrations (*C*_*0*_ = 50 and 100 mg/L), residuals of 5.550 and 16.540 mg/L were measured in the absence of biochar, which were higher values than those observed for low nonylphenol concentrations. In addition, the high nonylphenol concentrations had a slow initial phase of nonylphenol biodegradation, yielding 13.2% nonylphenol after the first two days, compared with 50.3% at low concentration; this may be due to the biotoxicity of nonylphenol [34], or it may be that enzyme saturation kinetics limited biodegradation at the higher concentrations used in these experiments [35]. Nonylphenol was degraded more at a biochar dosage of 0.005 g than in the absence of biochar, throughout the entire incubation period; for instance, 47.6 and 51.8% (*C*_*0*_ = 50 and 100 mg/L) nonylphenol was transformed in the initial two days, 125 and 99.2% higher than in respective experiments without biochar, indicating that biochar accelerated nonylphenol transformation. Interestingly, with the addition of more biochar, nonylphenol biodegradation was reduced. The results at both high and low nonylphenol concentrations were similar: degradation was inhibited at greater biochar dosage.

**Fig 2:**
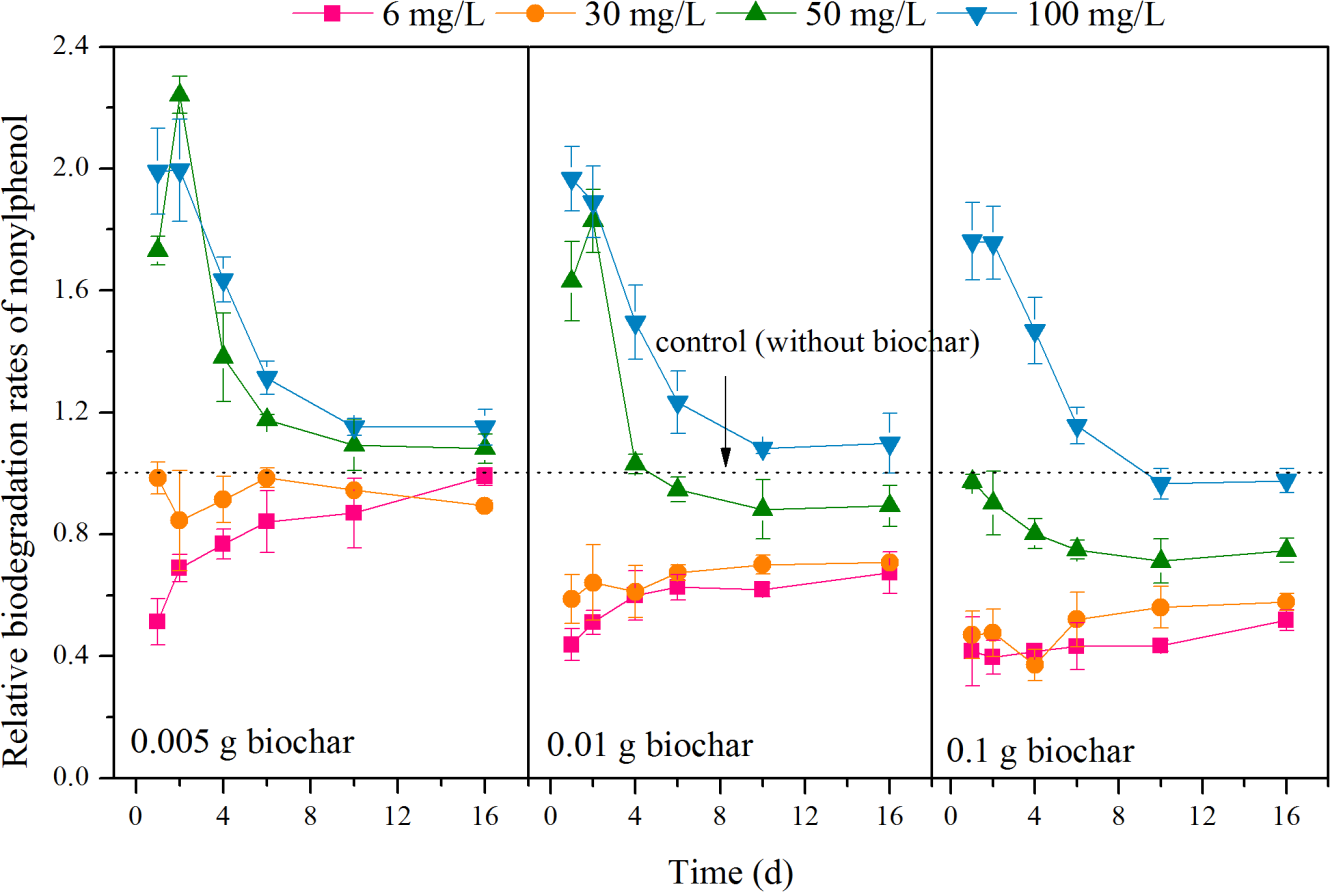
Relative rates of nonylphenol biodegradation with different biochar dosages. The ratios of nonylphenol biodegradation rates with and without biochar were calculated to obtain relative rates of biodegradation.

To further elucidate the relationship between adsorption and biodegradation of nonylphenol, the ratios of nonylphenol biodegradation rates with and without biochar were calculated to obtain relative rates of biodegradation, as shown in Fig 2. The relative rates of nonylphenol biodegradation were significantly affected by nonylphenol concentration (*P* < 0.01). For a 0.005 g biochar dosage, the relative rates varied between the low (*C*_*0*_ = 6 and 30 mg/L) and high (*C*_*0*_ = 50 and 100 mg/L) nonylphenol concentrations, with values less than 1.0 for low concentrations, showing a negative effect on biodegradation, and values greater than 1.0 for high concentrations, showing a positive effect. When the biochar dosage increased to 0.01 g, the negative effect persisted and became more pronounced at low concentrations (0.01 < *P* < 0.05). At the high nonylphenol concentration of 100 mg/L, the relative rate of biodegradation was still above 1.0, but at 50 mg/L, the rate decreased below 1.0 after 4 days, with a residual of 26.195 mg/L. At a 0.1 g biochar dosage, all relative rates were below 1.0, except at a nonylphenol concentration of 100 mg/L, for which the relative rate dropped below 1.0 after 10 days.

Overall, there was an appropriate biochar dosage range for optimal biodegradation at each given nonylphenol concentration: less than 0.01 g for 50 mg/L and 0.005–0.01 g for 100 mg/L. The promotion effect of biochar gradually slowed down as nonylphenol was degraded.

### Mechanism of biochar’s effect on nonylphenol biodegradation

To explore the mechanism of biochar’s effect on nonylphenol biodegradation at various concentrations, the change of aqueous (*C*_*aqueous*_) and solid phase (*C*_*solid*_) concentration were investigated during incubation (S1 and S2 Figs). In general, *C*_*aqueous*_ and *C*_*solid*_ both underwent processive reduction during incubation, similar to total nonlyphenol changing in Fig 1. On one hand, *C*_*aqueous*_ was much lower than the initial nonylphenol concentration without biochar addition, suggesting that the majority of nonylphenol was adsorbed to microorganisms, namely *C*_*solid*_, but did not significantly suppress biodegradation because the biological adhesion was unstable [35]. On the other hand, *C*_*aqueous*_ was evidently reduced in all biochar dosage treatments (*P* < 0.01) and became less available for microorganisms [32], leading to reduced biodegradability. Besides, *C*_*solid*_ was relatively greater at low initial concentrations (*C*_*0*_ = 6 and 30 mg/L) but getting lower at high initial concentrations (*C*_*0*_ = 50 and 100 mg/L) with biochar dosage getting larger, confirming the results suggested above that biochar addition played a inhibition role at low concentration of nonlyphenol, but showed promotion at high nonlyphenol concentrations. Furthermore, *C*_*aqueous*_ was higher at high nonylphenol concentrations than at low concentrations and thus may have acute toxicity against microorganisms [34], resulting in sluggish initial degradation.

**Fig 3:**
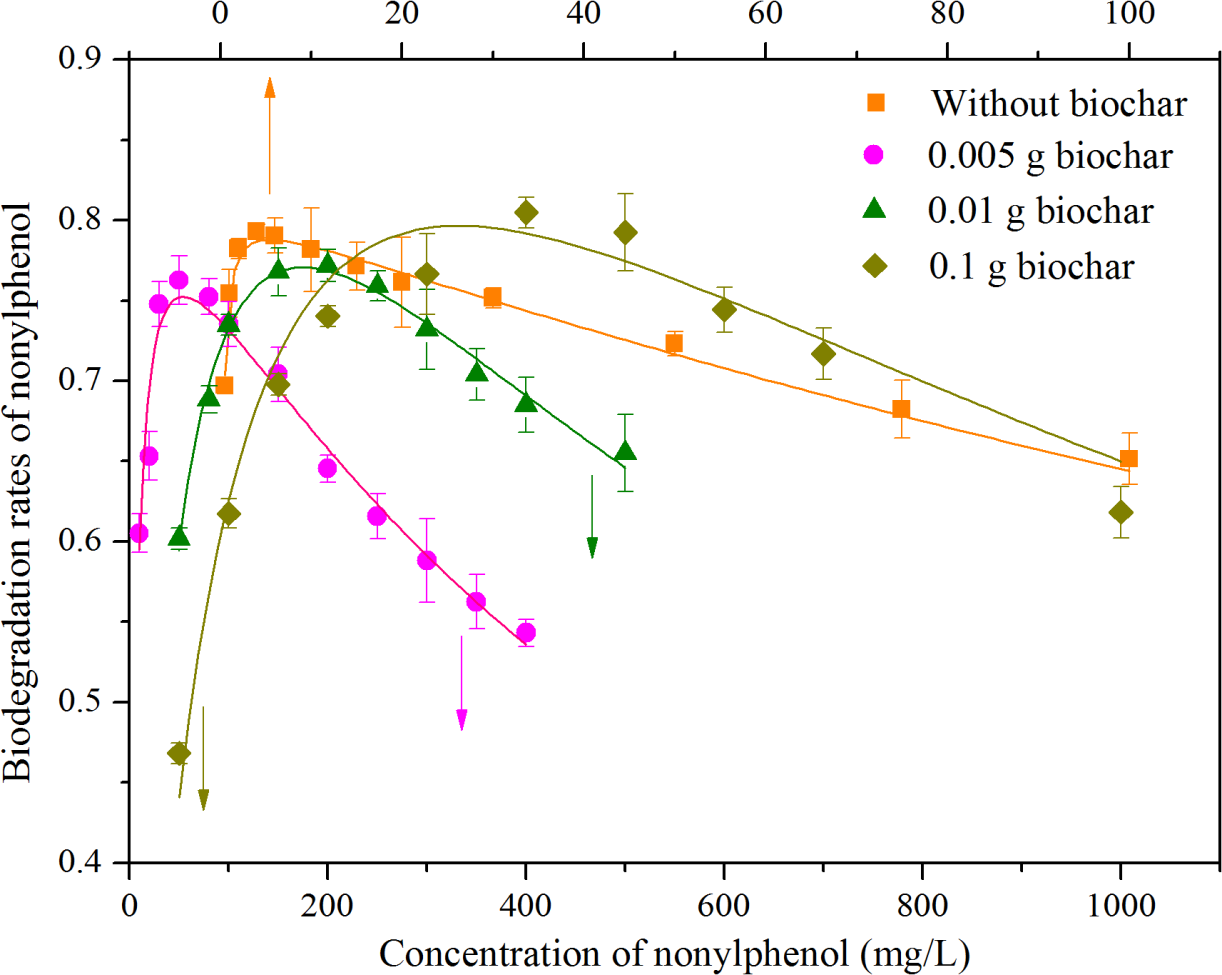
Concentration suppression curve of nonylphenol at different biochar dosages. The degradation rates of a series of nonlyphenol concentrations at 4 biochar dosages (0, 0.005, 0.01 and 0.1 g).

Concentration suppression curves of nonylphenol with 4 biochar dosages are shown in Fig 3. The optimal concentration in the absence of biochar was approximately 5.395 mg/L, so the degree of biodegradation at low nonylphenol concentrations (*C*_*0*_ = 6 and 30 mg/L) was slightly more complete than at higher concentrations (*C*_*0*_ = 50 and 100 mg/L). With the addition of biochar, all optimal concentrations gradually increased to 54.23, 175.22 and 332.35 mg/L for 0.005, 0.01 and 0.1 g biochar dosage, respectively. Among the low concentrations, biodegradation rates followed the progression *V*_*0*_ > *V*_*0*.*005*_ > *V*_*0*.*01*_ > *V*_*0*.*1*_, suggesting that nonylphenol transformation was inhibited by biochar addition. In addition, *V*_*0*.*005*_ reached a maximum at high nonylphenol concentration (*V*_*0*.*005*_ > *V*_*0*_ > *V*_*0*.*01*_ > *V*_*0*.*1*_ for *C*_*0*_ = 50 mg/L, *V*_*0*.*005*_ ≈ *V*_*0*.*1*_ > *V*_*0*_ > *V*_*0*.*1*_ for *C*_*0*_ = 100 mg/L), in accordance with results shown above. The reverse is also plausible if an applied nonylphenol concentration range corresponds with a specific biochar dosage. Therefore, the higher the nonylphenol concentration was, the more biochar required for remediation.

### Effects of biochar on form of sorbed nonylphenol

Previous studies have demonstrated that less than 40% of strongly immobilized nonylphenol could be released from sediment during 16 days of desorption [14]. Our results, however, showed rates of nonylphenol biodegradation up to 47.0–96.4% in the presence of biochar, implying that the resistant fractions were unexpectedly bioavailable. Based on the above results, desorption kinetics of residual nonylphenol after biodegradation, as well as changes in form of sorbed nonylphenol, including the rapid, slow and resistant fractions, were investigated.

**Fig 4:**
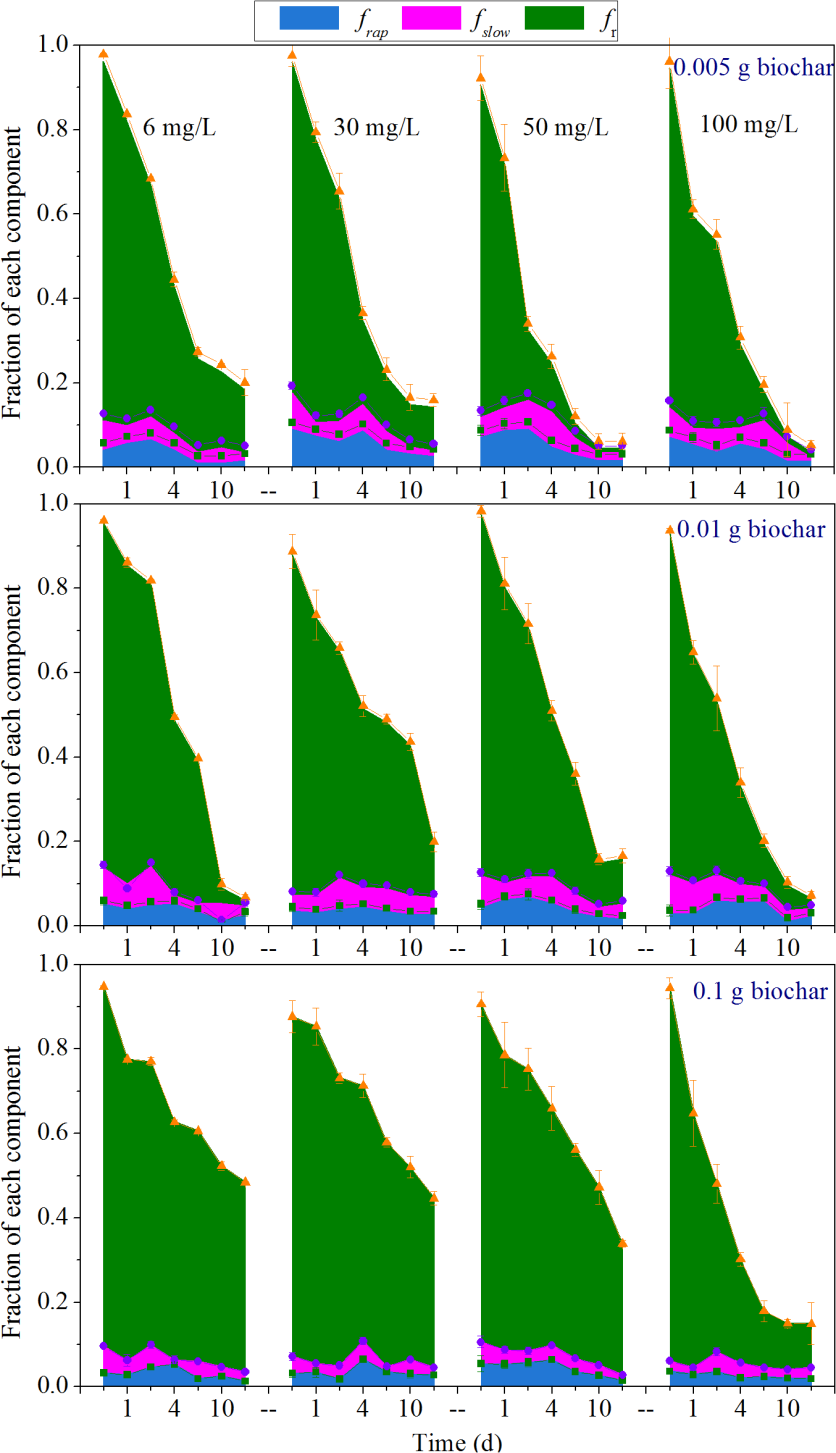
Fraction of each component during nonylphenol desorption kinetics experiments, fitted with transmutative modified two-domain model. Desorption data were fitted with a transmutative modified two-domain model, and each component gradually decreased over 16 d incubation time.

Statistical analysis revealed that the transmutative two-domain model provided a good fit for all biodegradation residual data (*R* ^*2*^ range 0.952–0.999). The changes in form of sorbed nonylphenol are shown in Fig 4.

Across experimental conditions, the desorption fractions followed the progression *f*_*r*_ > *f*_*rap*_ ≈ *f*_*slow*_ and were generally in order of 10^–2^ to 10^–1^, 10^–2^ and 10^–3^ to 10^–2^, respectively. Among these results, *f*_*r*_ was significantly greater than the other two fractions (*P* < 0.01), suggesting that *f*_*r*_ dominated in residual nonylphenol [14,36]. A slow decrease occurred in *f*_*rap*_, *f*_*slow*_ and *f*_*r*_ over the course of biodegradation experiments, representing the transformation of all three fractions. The values of *f*_*r*_ ranged from 0.852±0.0050 to 0.149±0.031, 0.782±0.0046 to 0.103±0.016, 0.788±0.053 to 0.0110±0.019, and 0.804±0.064 to 0.0120±0.011 for each nonylphenol concentration, respectively, with the addition of 0.005 g biochar; *f*_*r*_ underwent a significant decrease (*P* < 0.01), illustrating the biodegradability of *f*_*r*_. Furthermore, the minimal reduction of *f*_*r*_ at higher biochar dosage suggested the inhibition of the bioavailability of *f*_*r*_.

### Modeling biodegradable contributions from various desorption fractions

The results of correlation among degradable fractions during desorption demonstrated that the slopes for the degraded fraction and *f*_*rap*_+*f*_*slow*_ exceeded 1.0, indicating that apart from the rapid and slow fractions, there must be an additional contribution to biodegradation, namely *f*_*r*_.

Based on the above analysis, assumptions were put forward: First, all three fractions (rapid, slow and resistant) contributed independently to the total biodegradation, with independent biodegradation coefficients. Second, the rapid fraction could be degraded completely, and its coefficient was regarded as 1 (*BD*_*rap*_ = 1). Therefore, the overall biodegradable fractions may be described by the following relationships:

$$BD=BD_{rap}f_{rap}+BD_{slow}f_{slow}+BD_{r}f_{r}$$ (8)

*BD* represents the total biodegradable fraction, namely the rate of nonylphenol biodegradation in each period, and *BD*_*rap*_, *BD*_*slow*_ and *BD*_*r*_ represent the coefficients of the rapid, slow and resistant desorption fractions contributing to degradation. *f*_*rap*_, *f*_*slow*_ and *f*_*r*_ together with *BD* at each designated time interval were then subjected to statistical analysis using multiple linear regression to obtain the regression coefficients *BD*_*rap*_, *BD*_*slow*_ and *BD*_*r*_.

The calculated coefficients *BD*_*rap*_, *BD*_*slow*_ and *BD*_*r*_ at various desorption intervals are shown in Table 1. Data from all treatments were closely fitted by the model, and the independent variables explained 0.567–0.999 of the variations in the regression. All fitted *BD*_*slow*_ and *BD*_*r*_ values were above 0, confirming the bioavailability of the slow and resistant fractions. Specifically, the values of *BD*_*slow*_ ranged from 0.439±0.235 to 1.155±0.174, with most values either above 0.5 or very close to 1.0, suggesting good bioavailability of the slow desorption fraction. *BD*_*r*_ decreased to 0.01 upon increasing the biochar dosage to 0.1 g. The reduction of *BD*_*r*_ was due to intense suppression of bioavailability by biochar application [16]. In contrast, there was no apparent decrease in *BD*_*r*_ for the highest nonylphenol concentration (*C*_*0*_ = 100 mg/L), with all values exceeding 0.1, indicating the favorable biodegradability of the resistant fraction. Thus, given that higher nonylphenol concentrations are more easily degradable and that nonylphenol generates carbon and energy sources, the conditions were optimized for microbial growth, with the results demonstrating strong biological processes [29,30]. Based on the estimated *BD*_*rap*_, *BD*_*slow*_ and *BD*_*r*_, which were influenced by both nonylphenol concentration and biochar dosage, the rapid and slow fractions had favorable degradability, while the resistant fraction was only partly degraded. Nevertheless, the contributions of *f*_*r*_ could not be neglected due to the dominance of this parameter.

**Table 1:** Parameters obtained from fitting the measured biodegradation factor (*BD*) values to the multivariate model (Eq (8)).

Nonylphenol concentration (mg/L)	Biochar (g)	Fitted parameters			
		*BD*_*rap*_	*BD*_*slow*_	*BD*_*r*_	*R* ^*2*^
6	0.005	1	0.619±1.154	0.122±0.0993	0.862
	0.01	1	0.772±0.396	0.0513±0.0395	0.943
	0.1	1	0.635±0.0941	0.0106±0.00583	0.992
30	0.005	1	0.920±1.284	0.0669±0.132	0.567
	0.01	1	0.975±0.0900	0.0312±0.00845	0.996
	0.1	1	0.972±1.015	0.0179±0.0493	0.670
50	0.005	1	0.980±0.420	0.130±0.00522	0.999
	0.01	1	0.913±0.0569	0.0527±0.00539	0.998
	0.1	1	1.001±0.352	0.0213±0.0201	0.994
100	0.005	1	0.980±0.0420	0.130±0.00522	0.998
	0.01	1	1.037±0.114	0.117±0.00937	0.996
	0.1	1	1.155±0.174	0.131±0.0106	0.991

From the joint assessment of biodegradation and desorption, the following two conjectures were proposed: First, free aqueous phase nonylphenol was preferentially degraded [15], breaking the equilibrium between adsorption and desorption, promoting desorption of nonylphenol from biochar and converting the desorbed fractions into bioavailable form. Second, the microorganisms and nonylphenol were both adsorbed to biochar, allowing processes such as biofilm formation and the release of extracellular enzymes to contribute directly to the biodegradation of adsorbed nonylphenol [25,26]. As shown in Fig 4, *f*_*r*_ was still the major component in the system after 16 days of desorption, suggesting a limited extent of desorption for the resistant fraction [14]; thus, it was reasonable to conclude that the reduction of *f*_*r*_ was predominantly due to direct degradation by microorganisms.

### Coupled effects of biochar and microorganisms on the fate of HOCs

Typically, the application of biochar is coupled with microorganisms during HOCs remediation, resulting in a complex system in which adsorption-desorption of HOCs and microorganisms to biochar, abundant microbes and biofilms, and biodegradation of HOCs exist simultaneously. According to the results above, adsorbed HOCs were consumed gradually until becoming completely mineralized [21]. Most importantly, the effects of various biochar dosages vary, and the appropriate biochar dosage was capable of accelerating HOCs transformation. Contradictory results following biochar application in previous reports were attributed to the complex impact of HOCs concentration, biochar dosage and experimental time scale. At sites polluted with low concentrations of HOCs, biochar addition is inadvisable due to its inhibition of bioavailability; nonetheless, long-term biofilm formation may allow efficient in-situ treatment of HOCs contamination. Biochar plays a beneficial role in the transformation of high HOCs concentrations in the short term, but can inhibit transformation when HOCs are degraded gradually, similar to the effects at low HOC concentrations.

## Conclusion

In this study, nonylphenol was selected as a target contaminant and rice-straw biochar as an adsorbent to investigate the effects and mechanism of biochar dosage on nonylphenol biodegradation. The results showed that the optimal biochar dosage varied depending on the concentration of nonylphenol. Furthermore, three form of sorbed nonylphenol (*f*_*r*_, *f*_*rap*_ and *f*_*slow*_) decreased gradually, and the results of model quantification suggested that the slow and resistant fractions were subject to biodegradation dependent on the nonylphenol concentration and biochar dosage. Above all, biochar dosage is a very important factor that should be taken into account when biochar is applied to environmental remediation.

## Supporting Information

S1 Fig Changes in aqueous concentration of nonylphenol at 4 biochar dosages. The aqueous concentration of nonylphenol (6, 30, 50 and 100 mg/L) at each sampling time underwent a gradual reduction for 4 biochar dosages (0, 0.005, 0.01 and 0.1 g).
(TIF)

S2 Fig Changes in solid concentration of nonylphenol at 4 biochar dosages. The solid concentration of nonylphenol (6, 30, 50 and 100 mg/L) at each sampling time underwent a gradual reduction for 4 biochar dosages (0, 0.005, 0.01 and 0.1 g).
(TIF)

## References

[1] Beesley L, Moreno-Jimenez E, Gomez-Eyles JL, Harris E, Robinson B, Sizmur T (2011) A review of biochars' potential role in the remediation, revegetation and restoration of contaminated soils. Environmental Pollution 159: 3269–3282. 10.1016/j.envpol.2011.07.023 21855187

[2] Chen B, Chen Z (2009) Sorption of naphthalene and 1-naphthol by biochars of orange peels with different pyrolytic temperatures. Chemosphere 76: 127–133. 10.1016/j.chemosphere.2009.02.004 19282020

[3] Cheng G, Zhu L, Sun M, Deng J, Chen H, Xu X, et al (2013) Desorption and distribution of pentachlorophenol (PCP) on aged black carbon containing sediment. Journal of Soils and Sediments 14: 344–352.

[4] Gai X, Wang H, Liu J, Zhai L, Liu S, Ren T, et al (2014) Effects of feedstock and pyrolysis temperature on biochar adsorption of ammonium and nitrate. PLoS ONE 9(12): e113888 10.1371/journal.pone.0113888 25469875 PMC4254611

[5] Park JH, Choppala GK, Bolan NS, Chung JW, Chuasavathi T (2011) Biochar reduces the bioavailability and phytotoxicity of heavy metals. Plant and soil 348: 439–451.

[6] Ren XM, Chen CL, Nagatsu M, Wang XK (2011) Carbon nanotubes as adsorbents in environmental pollution management: A review. Chemical Engineering Journal 170: 395–410.

[7] Kukkonen JV, Mitra S, Landrum PF, Gossiaux DC, Gunnarsson J, Weston D (2005) The contrasting roles of sedimentary plant‐derived carbon and black carbon on sediment‐spiked hydrophobic organic contaminant bioavailability to Diporeia species and Lumbriculus variegatus. Environmental toxicology and chemistry 24: 877–885. 15839562 10.1897/04-171r.1

[8] Tatarkova V, Hiller E, Vaculik M (2013) Impact of wheat straw biochar addition to soil on the sorption, leaching, dissipation of the herbicide (4-chloro-2-methylphenoxy)acetic acid and the growth of sunflower (Helianthus annuus L.). Ecotoxicology and Environmental Safsty 92: 215–221. 10.1016/j.ecoenv.2013.02.005 23474069

[9] Smith JL, Collins HP, Bailey VL (2010) The effect of young biochar on soil respiration. Soil Biology and Biochemistry 42: 2345–2347.

[10] Gurwick NP, Moore LA, Kelly C, Elias P (2013) A systematic review of biochar research, with a focus on its stability in situ and its promise as a climate mitigation strategy. PLoS One 8: e75932 10.1371/journal.pone.0075932 24098746 PMC3786913

[11] Luo L, Lou LP, Cui XY, Wu BB, Hou JA, Xun B, et al (2011) Sorption and desorption of pentachlorophenol to black carbon of three different origins. Journal of Hazardous Materials 185: 639–646. 10.1016/j.jhazmat.2010.09.066 20971557

[12] Martin SM, Kookana RS, Van Zwieten L, Krull E (2012) Marked changes in herbicide sorption–desorption upon ageing of biochars in soil. Journal of Hazardous Materials 231: 70–78. 10.1016/j.jhazmat.2012.06.040 22795590

[13] Marchal G, Smith KE, Rein A, Winding A, de Jonge LW, Trapp S, et al (2013) Impact of activated carbon, biochar and compost on the desorption and mineralization of phenanthrene in soil. Environmental Pollution 181: 200–210. 10.1016/j.envpol.2013.06.026 23871817

[14] Liping L, Guanghuan C, Jingyou D, Mingyang S, Huanyu C, Qiang Y, et al (2014) Mechanism of and relation between the sorption and desorption of nonylphenol on black carbon-inclusive sediment. Environmental Pollution 190: 101–108. 10.1016/j.envpol.2014.03.027 24735684

[15] Alexander M (2000) Aging, bioavailability, and overestimation of risk from environmental pollutants. Environmental Science & Technology 34: 4259–4265.

[16] Chai YZ, Currie RJ, Davis JW, Wilken M, Martin GD, Fishman VN, et al (2012) Effectiveness of Activated Carbon and Biochar in Reducing the Availability of Polychlorinated Dibenzo-p-dioxins/Dibenzofurans in Soils. Environmental Science & Technology 46: 1035–1043. 10.1016/10.1021/es2029697 22136630

[17] Muter O, Berzins A, Strikauska S, Pugajeva I, Bartkevics V, Dobele G, et al (2014) The effects of woodchip- and straw-derived biochars on the persistence of the herbicide 4-chloro-2-methylphenoxyacetic acid (MCPA) in soils. Ecotoxicology and Environmental Safety 109: 93–100. 10.1016/j.ecoenv.2014.08.012 25173744

[18] Xin J, Liu X, Liu W, Zheng X (2014) Effects of biochar-BDE-47 interactions on BDE-47 bioaccessibility and biodegradation by Pseudomonas putida TZ-1. Ecotoxicology and Environmental Safety 106: 27–32. 10.1016/j.ecoenv.2014.04.036 24836874

[19] Meynet P, Hale SE, Davenport RJ, Cornelissen G, Breedveld GD, Werner D (2012) Effect of Activated Carbon Amendment on Bacterial Community Structure and Functions in a PAH Impacted Urban Soil. Environmental Science & Technology 46: 5057–5066. 10.1021/es2043905 22455603 PMC3342763

[20] Zhang QZ, Dijkstra FA, Liu XR, Wang YD, Huang J, Lu N (2014) Effects of biochar on soil microbial biomass after four years of consecutive application in the north China Plain. PLoS One 9: e102062 10.1371/journal.pone.0102062 25025330 PMC4098902

[21] Tong H, Hu M, Li FB, Liu CS, Chen MJ (2014) Biochar enhances the microbial and chemical transformation of pentachlorophenol in paddy soil. Soil Biology & Biochemistry 70: 142–150.

[22] Yang Y, Sheng G (2003) Enhanced pesticide sorption by soils containing particulate matter from crop residue burns. Environmental Science & Technology 37: 3635–3639. 12953876 10.1021/es034006a

[23] Farrell M, Kuhn TK, Macdonald LM, Maddern TM, Murphy DV, Hall PA, et al (2013) Microbial utilisation of biochar-derived carbon. Science of the Total Environment 465: 288–297. 10.1016/j.scitotenv.2013.03.090 23623696

[24] Sopena F, Bending GD (2013) Impacts of biochar on bioavailability of the fungicide azoxystrobin: a comparison of the effect on biodegradation rate and toxicity to the fungal community. Chemosphere 91: 1525–1533. 10.1016/j.chemosphere.2012.12.031 23478123

[25] Mercier A, Wille G, Michel C, Harris-Hellal J, Amalric L, Morlay C, et al (2013) Biofilm formation vs. PCB adsorption on granular activated carbon in PCB-contaminated aquatic sediment. Journal of Soils and Sediments 13: 793–800.

[26] Mercier A, Joulian C, Michel C, Auger P, Coulon S, Amalric L, et al (2014) Evaluation of three activated carbons for combined adsorption and biodegradation of PCBs in aquatic sediment. Water Research 59: 304–315. 10.1016/j.watres.2014.04.021 24813338

[27] Tanghe T, Dhooge V, Verstraete W (1999) Isolation of a bacterial strain able to degrade branched nonylphenol. Applied and Environmental Microbiology 65: 746–751. 9925611 10.1128/aem.65.2.746-751.1999 PMC91090

[28] Toyama T, Murashita M, Kobayashi K, Kikuchi S, Sei K, Tanaka Y, et al (2011) Acceleration of Nonylphenol and 4-tert-Octylphenol Degradation in Sediment by Phragmites australis and Associated Rhizosphere Bacteria. Environmental Science & Technology 45: 6524–6530. 10.1021/es201061a 21736332

[29] Wang Z, Yang Y, Sun W, Xie S (2014) Biodegradation of nonylphenol by two alphaproteobacterial strains in liquid culture and sediment microcosm. International Biodeterioration & Biodegradation 92: 1–5.

[30] De Weert J, Viñas M, Grotenhuis T, Rijnaarts H, Langenhoff A (2010) Aerobic nonylphenol degradation and nitro-nonylphenol formation by microbial cultures from sediments. Applied Microbiology and Biotechnology 86: 761–771. 10.1007/s00253-009-2394-9 20043151 PMC2825322

[31] Liu J, Shan J, Jiang B, Wang L, Yu B, Chen J, et al (2014) Degradation and bound-residue formation of nonylphenol in red soil and the effects of ammonium. Environmental Pollution 186: 83–89. 10.1016/j.envpol.2013.11.017 24368312

[32] Kong LL, Liu WT, Zhou QX (2014) Biochar: an effective amendment for remediating contaminated soil. Reviews of Environmental Contamination and Toxicology 228: 83–99. 10.1007/978-3-319-01619-1_4 24162093

[33] Xu RK, Xiao SC, Yuan JH, Zhao AZ (2011) Adsorption of methyl violet from aqueous solutions by the biochars derived from crop residues. Bioresource Technology 102: 10293–10298. 10.1016/j.biortech.2011.08.089 21924897

[34] Mariel AC, Alejandra BP, Silvia PCC (2014) Developmental toxicity and risk assessment of nonylphenol to the South American toad, Rhinella arenarum. Environmental Toxicology and Pharmacology 38: 634–642. 10.1016/j.etap.2014.08.014 25195099

[35] Writer JH, Barber LB, Ryan JN, Bradley PM (2011) Biodegradation and attenuation of steroidal hormones and alkylphenols by stream biofilms and sediments. Environmental Science & Technology 45: 4370–4376. 10.1021/es2000134 21520955

[36] Rhodes AH, Riding MJ, McAllister LE, Lee K, Semple KT (2012) Influence of activated charcoal on desorption kinetics and biodegradation of phenanthrene in soil. Environmental Science & Technology 46: 12445–12451. 10.1021/es3025098 23092507

