# Supplementary figures and images for: Application of Rice-Straw Biochar and Microorganisms in Nonylphenol Remediation: Adsorption-Biodegradation Coupling Relationship and Mechanism
Source: PLoS One. 2015 Sep 8;10(9):e0137467. doi: 10.1371/journal.pone.0137467 (PMC4562627; doi:10.1371/journal.pone.0137467)

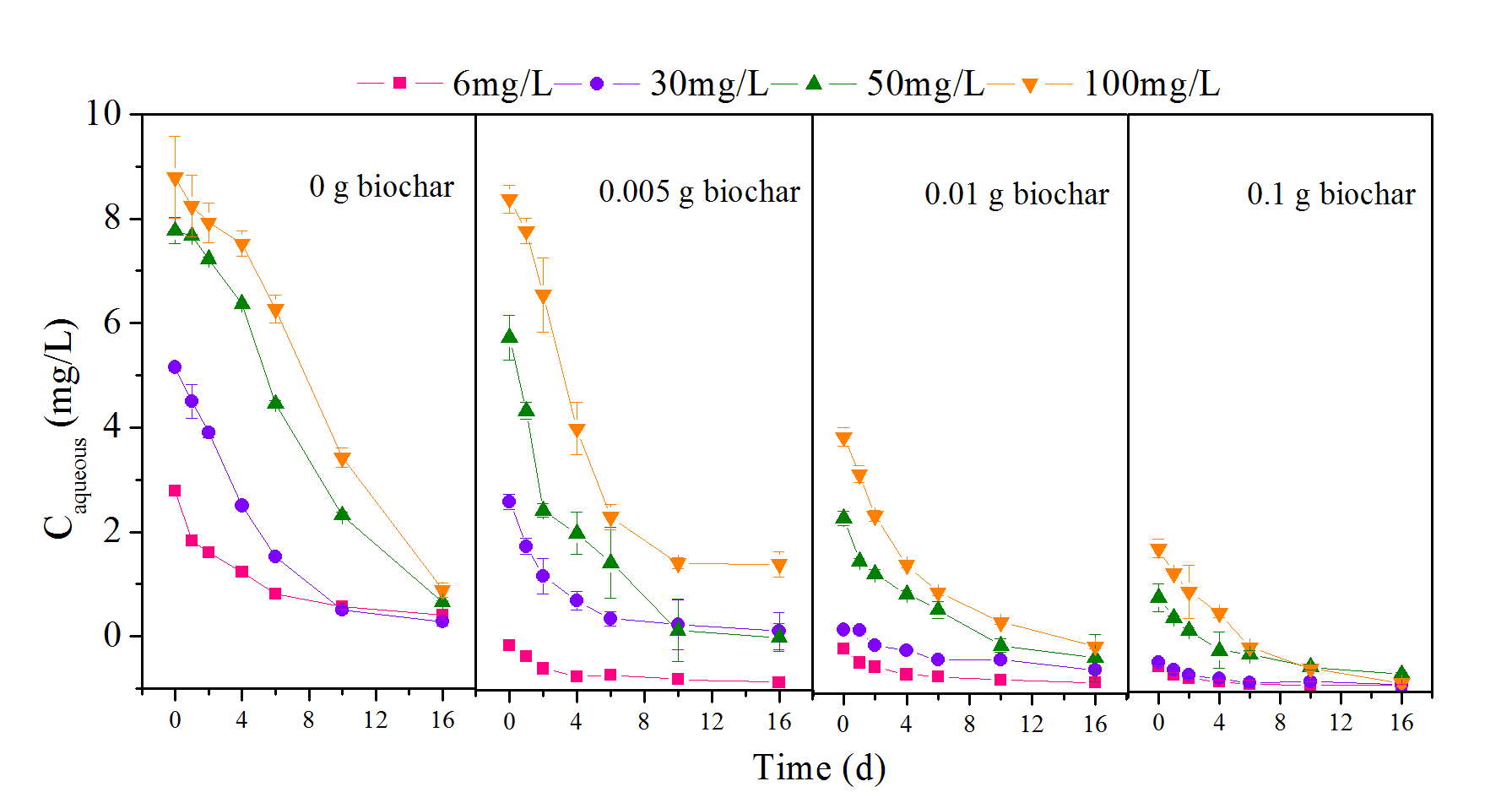

Supplement: S1 Fig — The aqueous concentration of nonylphenol (6, 30, 50 and 100 mg/L) at each sampling time underwent a gradual reduction for 4 biochar dosages (0, 0.005, 0.01 and 0.1 g). (TIF) [file pone.0137467.s001.tif]

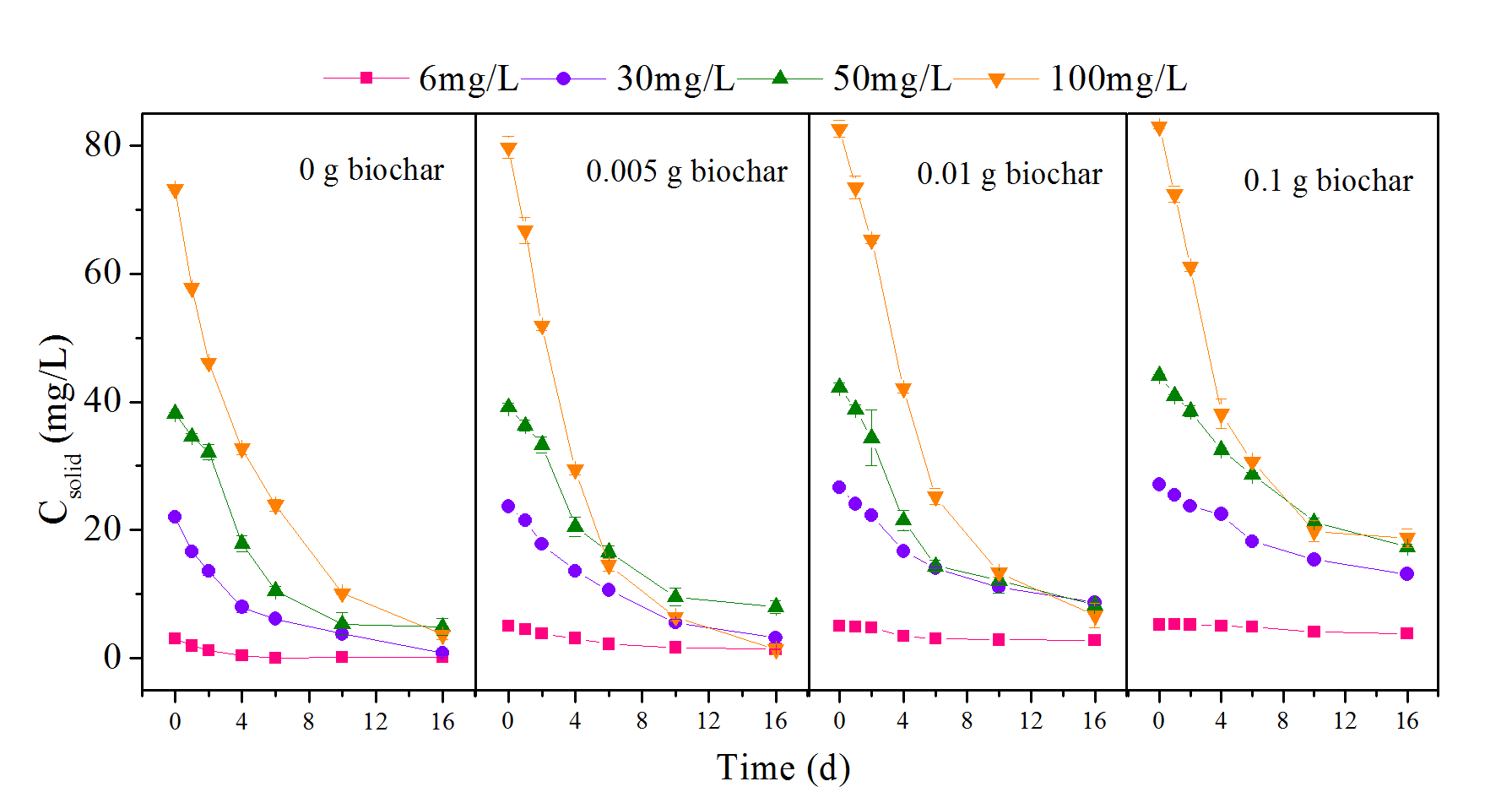

Supplement: S2 Fig — The solid concentration of nonylphenol (6, 30, 50 and 100 mg/L) at each sampling time underwent a gradual reduction for 4 biochar dosages (0, 0.005, 0.01 and 0.1 g). (TIF) [file pone.0137467.s002.tif]
